# A linear bi-level multi-objective program for optimal allocation of water resources

**DOI:** 10.1371/journal.pone.0192294

**Published:** 2018-02-14

**Authors:** Ijaz Ahmad, Fan Zhang, Junguo Liu, Muhammad Naveed Anjum, Muhammad Zaman, Muhammad Tayyab, Muhammad Waseem, Hafiz Umar Farid

**Affiliations:** 1 Centre of Excellence in Water Resources Engineering, University of Engineering and Technology, Lahore, Pakistan; 2 Key Laboratory of Tibetan Environment Changes and Land Surface Processes, Institute of Tibetan Plateau Research, University of Chinese Academy of Sciences, Beijing, China; 3 CAS Center for Excellence in Tibetan Plateau Earth Sciences, Beijing, China; 4 University of Chinese Academy of Sciences, Beijing, China; 5 School of Environmental Science and Engineering, South University of Science and Technology of China, Shenzhen, China; 6 State Key Laboratory of Cryospheric Science, Northwest Institute of Eco-Environment and Resources, Chinese Academy of Sciences, Lanzhou, P.R. China; 7 Research Center of Fluid Machinery Engineering & Technology, Jiangsu University, Zhenjiang, China; 8 College of Hydraulic and Environmental Engineering, China, Three Gorges University, Yichang, China; 9 Department of Agricultural Engineering, Bahauddin Zakariya University, Multan, Pakistan; Southwest University, CHINA

## Abstract

This paper presents a simple bi-level multi-objective linear program (BLMOLP) with a hierarchical structure consisting of reservoir managers and several water use sectors under a multi-objective framework for the optimal allocation of limited water resources. Being the upper level decision makers (i.e., leader) in the hierarchy, the reservoir managers control the water allocation system and tend to create a balance among the competing water users thereby maximizing the total benefits to the society. On the other hand, the competing water use sectors, being the lower level decision makers (i.e., followers) in the hierarchy, aim only to maximize individual sectoral benefits. This multi-objective bi-level optimization problem can be solved using the simultaneous compromise constraint (SICCON) technique which creates a compromise between upper and lower level decision makers (DMs), and transforms the multi-objective function into a single decision-making problem. The bi-level model developed in this study has been applied to the Swat River basin in Pakistan for the optimal allocation of water resources among competing water demand sectors and different scenarios have been developed. The application of the model in this study shows that the SICCON is a simple, applicable and feasible approach to solve the BLMOLP problem. Finally, the comparisons of the model results show that the optimization model is practical and efficient when it is applied to different conditions with priorities assigned to various water users.

## Introduction

The ever-increasing population growth and industrialization are putting constant pressure on water resources and it is more likely that the available water resources may not be able to meet the future water demands. The shortage of water resources has become more severe due to the uneven distribution of available water resources among various water demand sectors and is a major constraint to economic development in many countries around the world [1]. Conflicts among various water demand sectors often arise when these sectors compete for limited water resources. As a solution to these conflicts, earlier studies have developed optimization models for water allocation to achieve sustainable development, such as dynamic programming [2,3], genetic algorithms [4,5], and game theory approach [6]. However, these models are difficult to apply to practical water allocation issues because of their complex programming requirements to deal with discontinuous, multi-dimensional, non-differentiable, stochastic, uncertainty and non-convexity problems in solving multi-objective functions [7,8].

In most practical cases, the major problem is the allocation of sustainable water to various water demand sectors, such as irrigation, industry, domestic and environment. Single objective programming has been extensively used in resolving water allocation conflicts of competing water demand sectors [9–14]. However, models using the single objective function are not able to provide a sustainable water allocation program [15]. Most of these models aim to maximize the economic returns to various water users and do not consider the satisfaction rate (ratio of the amount of water supplied to the normal demand of a particular water user) for a sustainable water allocation system. Thus, the researchers have turned to multi-objective programming.

As long as the available water is more than the demands of different water demand sectors, all users can coexist without conflicts and as such the problem of water allocation does not arise [16]. But this is not the case; increased water demands often intensify the conflicts among different water users. Therefore, to avoid the present as well as future conflicts between the competing water users, researchers and scientists have given more emphasis on developing tools and techniques for sustainable water resources management. Due to its multidisciplinary nature and complexity, multi-objective programming has frequently been applied to resolve water related conflicts [17–19] such as water allocation [20]. Babel et al. [16] developed a multi-objective water allocation model using the SICCON technique to support reservoir operators and managers in optimizing water allocation for a hypothetical reservoir. Using multi-objective programming and rainfall forecasts, Khummongkol et al. [21] developed a multi-objective integrated model for optimizing water allocation and management so as to maximize the NEB. By considering the water resources security, Wang et al. [22] developed a multi-objective water allocation model to improve the eco-environmental and socio-economic benefits in Zhangjiakou region of northern China. Roozbahani et al. [23] successfully applied a multi-objective optimization program to develop the altered water allocation strategies for resolving the water conflicts of various demand sectors. Using the compromised constraint technique, Roozbahani et al. [24] developed a multi-objective optimal water allocation model to resolve the water conflict among competing water demand sectors by simultaneously addressing economic, social and environmental aspects. Furthermore, several researchers explored and discussed conflicts and remedial measures that can generate efficient, sustainable and equitable water allocation practices [25–28].

However, in many countries including Pakistan, there are two different groups of water use sectors and water managers in the water resources allocation system. Hence, the conflicts are not only among various water use sectors but also between water use sectors and water managers. Therefore, in the planning of a water allocation program, different water users and reservoir planners/managers can have different objectives thereby resulting in incommensurable conflicts [29]. For these cases, when the managers need to make a choice among multi-profits, traditional mathematical programming cannot deal with such dilemma under a hierarchical structure, a bi-level approach within multi-objective framework must be used to carry out the different profit preference analysis [30]. Multi-level and bi-level programming was first introduced by Candler and Norton [31] which is a mathematical programming technique to solve decentralized planning problems with multiple decision makers in a multi-level or hierarchical structure. This decision making process is extremely practical to the decentralized systems, such as agriculture, economic systems, water resources planning, and particularly for conflict resolution [32].

Several bi-level optimization techniques are available to deal with water allocation problems under a hierarchical structure of upper level DMs (i.e. leaders) and lower level DMs (i.e. followers). Ling and Gui [33] developed a bi-level program for the optimal allocation of water resources in the Jiangxi province of China by using genetic algorithms and simplex method for solving the upper level and lower level programming problems, respectively. Lv et al. [34] developed an interval fuzzy bi-level programming (IFBP) approach which has played an extremely important role in solving bi-level water resources allocation problems, with the DMs in a hierarchical structure conflicting with each other.

By balancing the degree of satisfaction between the upper level and lower level DMs in optimization of the equitable water distribution, Xu et al. [35] developed a bi-level programming model with fuzzy random variables in solving the regional water resources allocation problem. Fang et al. [30] used the fuzzy goal programming approach to address the bi-level water allocation problem in Wuwei basin of China. In their program, the upper level decision makers were solved first, and then used as the tolerance to solve the lower level decision makers. Guo et al. [36] developed a bi-level optimization model that allocates water resources rationally to different water users, and prevents overexploitation by optimizing the social, economic, agricultural, environmental and groundwater preservation benefits in the Hebei Province of China. Wei and Hu [37] developed a bi-level model to solve the water allocation problem in Qujiang River basin in China so as to provide equality, stability and economic efficiency for sustainable water resources development. Hu et al. [38] developed a multi-objective bi-level model for Qujiang River basin of China with the upper level DMs reflecting the river basin authority’s allocation principle of equity and stability and the lower level DMs aimed at ensuring maximal economic benefit efficiency for each subarea. In other studies, different methods such as Stackelberg game theory, particle swarm optimization and artificial neural network have been used to solve the bi-level decision making problems in water exchange in eco-industrial parks [39], manufacturer-retailer supply chain problems [40], and lot-sizing problems [41], respectively. However, these techniques have seldom been applied to practical cases for the allocation of water resources because of their complexity.

In developing countries including Pakistan, reservoir operators are not well trained to adopt and implement the advanced, complex and stochastic techniques for reservoir operation. Simonovic [42] discussed the limitations and remedial measures of reservoir operation techniques to make them more convenient and acceptable to the reservoir operators and managers. Many researchers [43–46] have acknowledged that the necessarily abstract nature of complicated reservoir operation optimization models resulted in their limited application and use. Reservoir operators and managers may feel uncomfortable while applying complicated optimization techniques; the stochastic nature of hydrologic variables made them even more complex [47,48].

Therefore, using a SICCON technique, this study aims to optimally allocate the available water resources among competing water users by developing a simple and applicable BLMOLP with multiple level decision makers and obtaining solutions that can optimize both the total benefits to the society and the economic benefits of each water use sector. In addition, different scenarios were developed to evaluate the model applicability and to assist decision makers in formulating solutions that are flexible and can satisfy various future conditions.

### Study area

**Fig 1:**
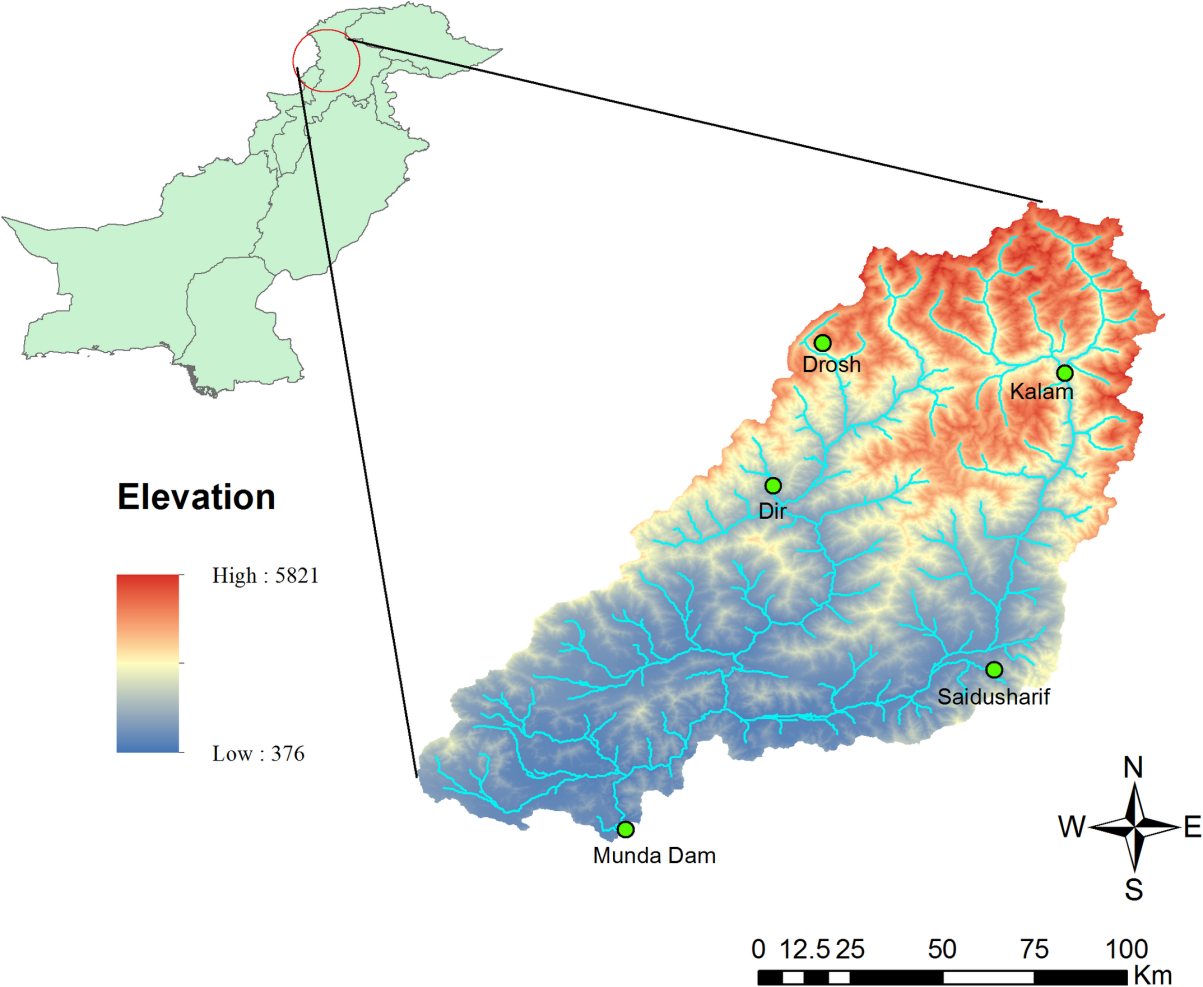
Location map of Swat River basin.

Swat River is one of the main rivers in Khyber Pakhtunkhwa province of Pakistan with a drainage area of 13,650 km^2^ at Munda Dam site. Upper Swat and Panjkhora rivers are two major tributaries of Swat River Basin. The general flow direction of the main river and its tributaries is from north to south, as shown in Fig 1. At Kalam, Gabral and Ushu rivers join to form upper Swat River. These rivers originate from the high mountains of Swat Kohistan, where the mean elevation is 4,500 m. The basin elevation falls gradually from 4,500 m to 910 m downstream of Kalam and the valley is also wider up to Chakdara. Originating from the high hills of Dir Kohistan, Panjkhora River joins upper Swat River near Kulangi about 41.0 km downstream of Chakdara gauging station. From Kulangi post down to the proposed Munda dam, the river flows through a narrow gorge, which is about 55.0 km long.

Munda dam is a multipurpose dam, which is under construction with an effective storage capacity of 815 Mm^3^. The water budget in Swat River has a surplus during the wet season (Apr-Sep) and a deficit during the dry season (Oct-Mar). The mean annual discharge in the Swat River is an estimated 337 (m^3^/s of which more than 82% of the runoff is in the wet season (Apr-Sep) due to the monsoon rain. Hence, Swat River is a water scarce basin during the dry season and the limited available water resources need to be managed efficiently for sustainable economic development. Further, the difference between the water supply and the water demand is increasing. To maximize individual sectoral benefits, water conflicts often arise among various water users competing for the limited available water resources. On the other hand, for the upper level decision makers (i.e. the leaders), it is more important to balance the economic benefits of each water demand sector, which is different from the objective of the lower level decision makers (i.e. followers) which is to maximize their own net benefits. Therefore, bi-level programming has been used for the optimization of water allocation in Swat River Basin.

The daily observed river flow data is available from 1964 to 2010 at Munda Dam site, which is obtained from the Surface Water Hydrology Project (SWHP) to analyze the variations in the monthly and annual river flows. As the Munda Dam site is situated along the main Swat River, the observed discharge data at the station have been used for the calibration of the hydrological model and validation of the modeled simulated flows.

**Fig 2:**
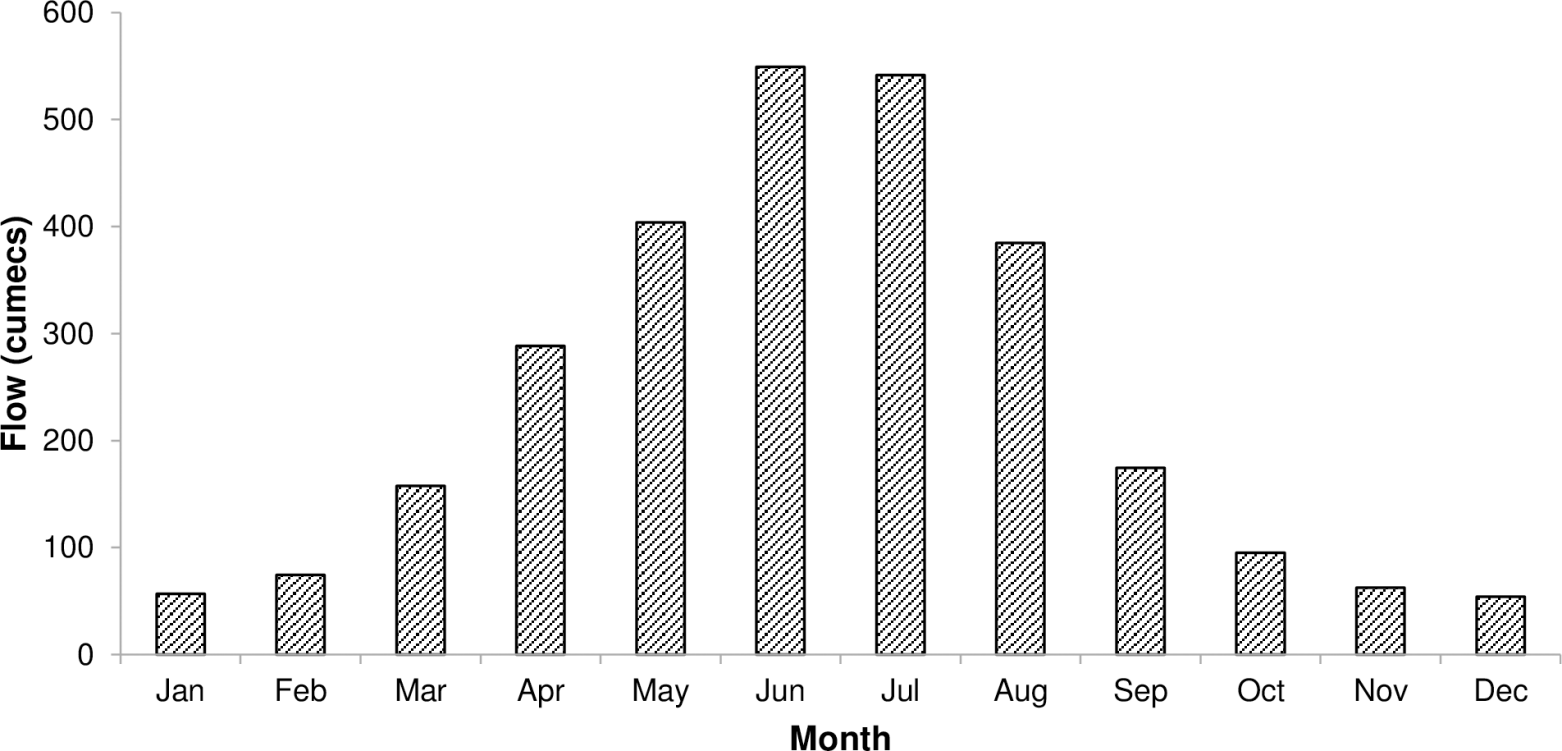
Mean monthly river flows at Munda dam during 1964–2010.

The mean monthly river flows at Munda Dam site from 1964 to 2010 are shown in Fig 2. The dam site receives the highest flows from May to August during to the monsoon season. On the other hand, the river flows are lowest from October to March. The Munda Dam site receives 66% of the total annual river discharge from May to August, and 82% from April to September (i.e. the wet season). Therefore, water allocation optimization is not required during the wet season as the available water resources are greater than the demand [16] and all the water users are fully satisfied. However, water use sectors compete for the limited available water in the dry season.

The water resources of the Swat River basin are being used to fulfill water demands for human sustenance and to meet the increasing needs of regional socio-economic development. In Swat River basin, the reservoir managers need to gain water entitlements from the river basin authority and thereafter distribute water to water use sectors. For water allocation, the reservoir managers generally consider domestic, agricultural, industrial, and environment sectors. The domestic sector covers residential and municipal needs. The industrial sector needs water for several purposes such as the production of chemicals, food, paper, and construction. Agricultural water is mainly used for irrigation and livestock, and the environmental sector guarantees the protection of river environment. Domestic and agricultural water demands are critical for life, while industrial water is essential for regional development. To satisfy the socioeconomic considerations, reservoir managers desire to maximize their economic benefit efficiency. All these sectors are included in the lower level objective functions.

## Materials and methods

### Conceptual framework

**Fig 3:**
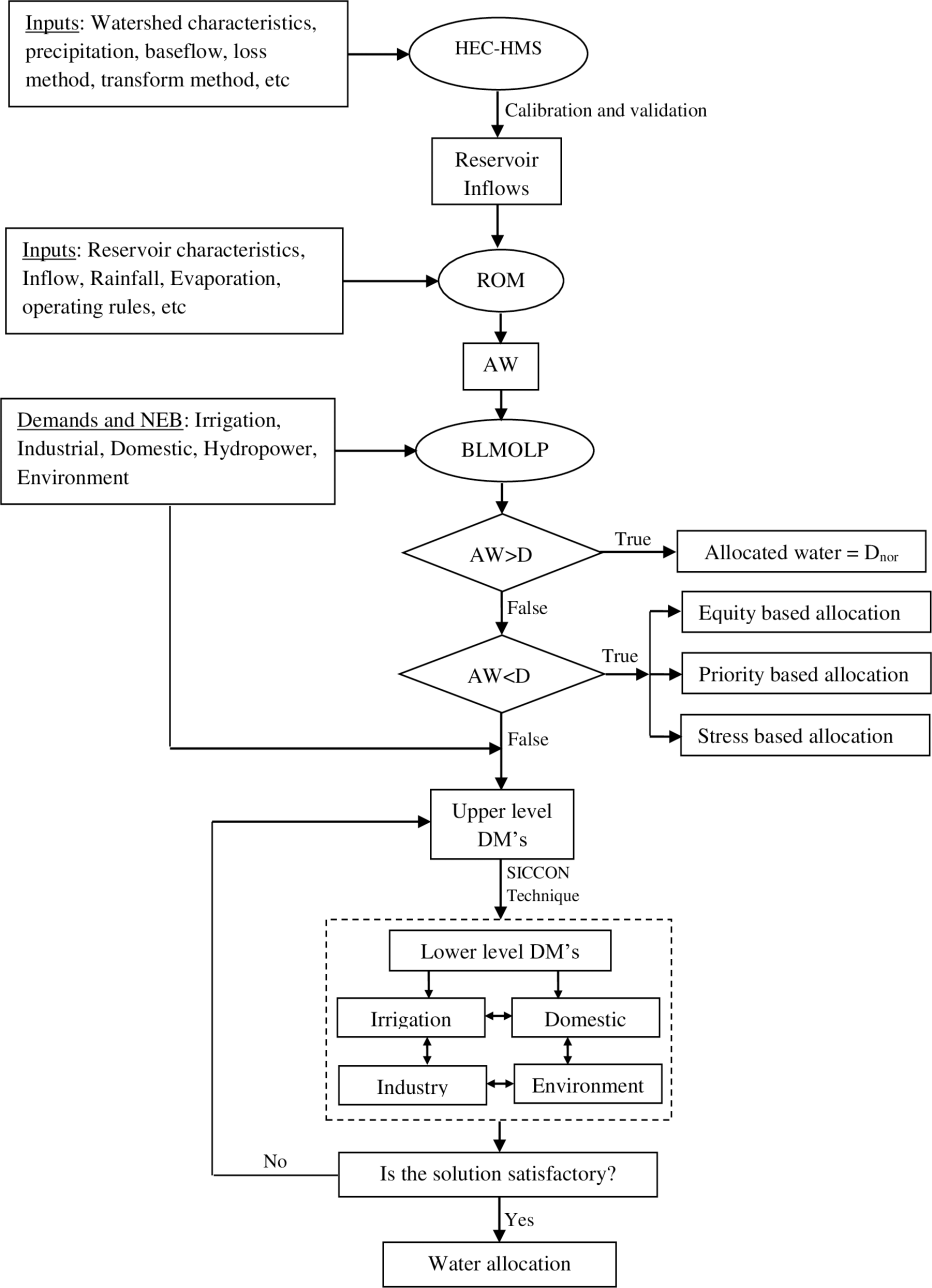
Basic working principle of bi-level multi-objective linear program (BLMOLP).

The basic working principle and components of the model are shown in Fig 3. The model consists of two components: the reservoir operation model (ROM) and a bi-level multi-objective linear program. Using the observed flows to calibrate HEC-HMS model developed by USACE-HEC [49], the inflows at different locations of the river have been estimated. After the verification of the estimated inflows, a standard reservoir operation algorithm has been developed in the Matlab language. In the algorithm, the reservoir physical characteristics and the inflows into the reservoir are the inputs and the output is the volume of the available water (AW), which is the input to BLMOLP. If the AW is greater than the normal demand (D_nor_) for all the considered water demand sectors, all the sectors get their full share of water and the optimal water allocation is not required as the upper level and lower level objectives are fully satisfied. However, this is not the usual case in most of the real-life world water allocation situations and water conflicts often arise among different competing sectors due to the shortage of AW.

When the AW is less than the minimum demand, the water can be allocated according to equity based, priority based or stress-based supply. The equity-based allocation is concerned with the fairness of the allocation which may or may not be consistent with the efficiency objectives which balance the different needs of multiple users and uses. The priority based allocation can either be user defined or doctrine based (i.e. the riparian doctrine or the prior-appropriation doctrine) [16]. The user defined priority can either be single or multiple priorities, in which the user has the privilege of making the decision. In the stress-based allocation, the criterion is to distribute the stress due to the deficiency of water equally among various water users. However, in the present case, as the total AW is greater than the minimum demand of each sector, and none of the sector faces water stress. Therefore, the stress-based water allocation criterion is not considered in this study.

Bi-level multi-objective linear programming has been used in the optimal water allocation based on the upper and lower level objective functions. The water allocation problem being considered in this study is a Stakelberg game with multiple objectives at the upper level, and a single objective at the lower level. For the upper level decision makers, they make their decisions based on a balance among the competing water demand sectors i.e. maximizing the rate of satisfaction and economic benefits, thereby maximizing the overall benefits from the water resources system. On the other hand, the lower level decision makers make their decisions based on maximizing their own individual sectoral benefits by receiving maximum amount of water. The upper level DMs set their goal and/or decisions and then based on the goal or decisions, each subordinate level of the system determine their optima independently. The decision of lower level DMs are then submitted and modified by the upper level DMs with consideration of the overall benefits for the water allocation system; the process is continued until a satisfactory solution is reached.

### Optimization techniques

The basic algorithm used for the optimal allocation of limited water resources to various sectors is based on the deterministic linear programming. Two optimization techniques, i.e. the weighting technique (WT) and simultaneous compromise constraint (SICCON) techniques, have been used to convert the upper level and lower level objectives into a multi-objective function.

#### Weighting technique (WT)

In this technique, different weights are assigned to the objective functions according to the degree of importance. By grouping the individual objectives into one single objective function, the upper level and lower level decision making problems becomes one single decision-making problem and is given by the following equation:

$$Z=G\times \left[\sum_{i=1}^{n}w_{i}\times z_{i}\right]$$ (1)

Where Z is the optimal allocation values, G is the minimization or maximization function, n is the number of objectives, and z is the individual objective function.

The existing analytical methods for the determination of weights may result in different values; this implies that the relative importance of different water demand sectors is indicated by the human feelings, which is quite subjective in nature. The transformation of human feelings regarding the relative importance of different sectors into numerical values of weights is quite difficult. However, in practical cases, it can be done by a group of decision makers and/or experts by distributing a questionnaire to each member of the group. This group of decision makers and/or experts maybe comprise of academicians, water scientists and managers who are involved in water resources development, planning and management projects. Each group member is asked to place a numerical value next to each objective according to their relative importance based on their knowledge and experience. The most important objective will get a value of 1.0, and the next most important objective’s value will be 2.0 and so on. In this context, ranking approach can be an appealing alternative. The numerical values placed by decision makers and/or expert are converted into scores, such as; for k objective functions, the rank 1 will be (k-1), and similarly rank 2 will be (k-2), and so on. These scores are converted to weights by the following relationship;

$$w_{m}=\frac{\sum_{j=1}^{n_{d}}e_{mj}}{\sum_{m=1}^{k}\sum_{j=1}^{n_{d}}e_{mj}}\Rightarrow m=1,2,3,\ldots \ldots ,n$$ (2)

Where, *e*_*mj*_ is the converted scores of the m^th^ objective given by the j^th^ expert; n_d_ is the number of judges in the group.

The upper-level DMs tends to distribute water based on equity system to achieve the maximum level of satisfaction of various water users for the sustainable socio-economic development of a society. However, the lower-level DMs tend to maximize individual sectoral benefits by allocating the maximum amount of water. The weights may be assigned to the upper or lower level DMs depending upon their importance in the society. For example, for a developed country the weightage given to upper-level DMs may be less as compared to lower-level DMs, whereas for a developing or underdeveloped country, the weightage given to lower-level DMs may be more than that of upper-level DMs. However, in this study, same weights are given to the upper- and lower- level DMs for simplicity.

#### Simultaneous compromise constraint (SICCON) technique

Simultaneous compromise constraint (SICCON) technique has been used to solve the bi-level problem for the water resources allocation. This technique is based on the compromise-constraint approach [50]. To find the optimal solution between the objectives of the upper level and lower level DMs, a compromise-constraint is added to the problem. The compromise-constraint forces the upper level and lower level DMs to be an equal weighted difference from the individual optimal solution. A single objective problem with the weighted sum of the original objective functions, subjective to the compromise-constraint plus the original ones, is solved. The compromise constraints are incorporated into each combination of decision making with two additional deviational variables representing the positive and negative deviations from the ideal or supposed to-be-zero values. Each deviational variable forms the compromise-constraint in the standard form, as follows:

$$\mathrm{Maximize}\;Z=\left[w_{u}\times f_{u}\left(x\right)+w_{l}\times f_{l}\left(x\right)\right]-\sum_{u\neq l}\left(\sigma_{ul}^{-}+\sigma_{ul}^{+}\right)$$ (3)

**Fig 4:**
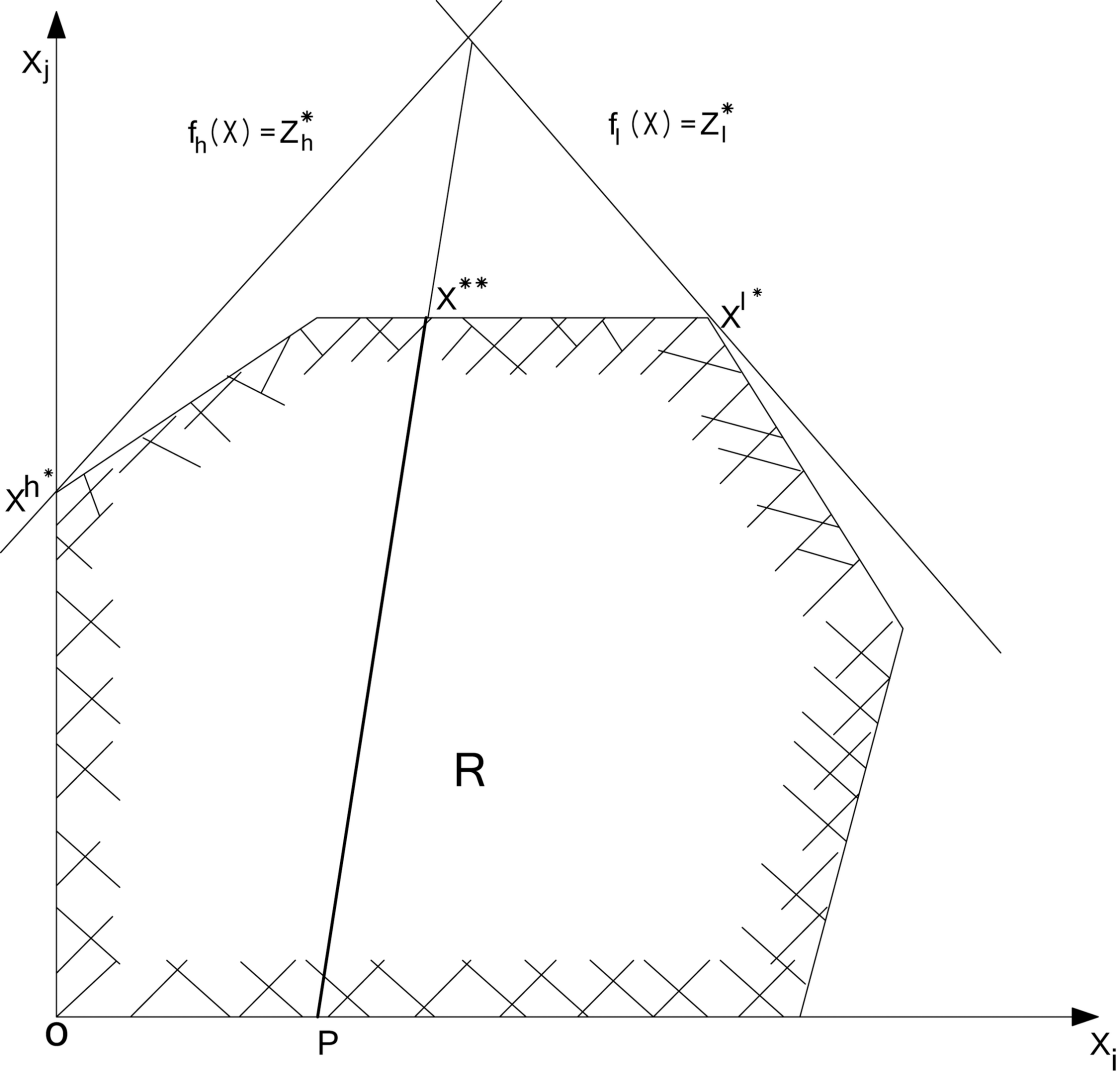
Graphical illustration of compromise constraint approach.

Subjected to

$$w_{u}[f_{u}(x)-z_{u}^{*}]-w_{l}[f_{l}(x)-z_{l}^{*}]+(\sigma_{ul}^{-}-\sigma_{ul}^{+})=0$$ (4)

where *f*_*u*_(*x*) and *f*_*u*_(*x*) are the upper level and the lower level objective functions whose individual maxima are $z_{u}^{*},$$z_{l}^{*}$, respectively, *w*_*u*_, *w*_*l*_ are the weights of the upper level and lower level DMs, respectively. The variables $\sigma_{ul}^{-},$ $\sigma_{ul}^{+}$ are the negative and positive deviations from the supposed to-be-zero (ideal solution) values of the compromise constraint between the individual DMs $z_{u}^{*}$ and$z_{l}^{*}$, respectively. The introduction of $\sigma_{ul}^{-},$ $\sigma_{ul}^{+}$ is necessary to account for the non-zero values on the left-hand side of the compromise-constraint. The variable $\sigma_{ul}^{-}$ is non-zero if the left-hand side of the constraint is negative; $\sigma_{ul}^{+}$is non-zero if the value is positive; and for an ideal solution both $\sigma_{ul}^{-}$ and $\sigma_{ul}^{+}$ should be zero. Therefore, both positive and negative deviational variables ($\sigma_{ul}^{-},$$\sigma_{ul}^{+}$) need to be minimized, which lead to the mutual exclusiveness of $\sigma_{ul}^{-}$ and $\sigma_{ul}^{+}$. The basic theory of the compromise constraint approach for bi-level decision making is shown in Fig 4.

As shown in the Fig 4, the point of intersection of the decision makers’ *f*_*u*_*(x)* and *f*_*l*_*(x)* does not fall within the feasible region (R). Hence, the two DMs have to move inside R until the intersection point is within their common region. In other words, the solution to the compromise-constraint lies on the line X** in Fig 4.

In order to solve the above mentioned problem, it is necessary to introduce the concept of a unique and efficient solution. The most logical way of doing this is to avail the maximization of the weighted sum of all objectives functions and thus coming up with an objective function which comprises of two parts: the weighted sum of all objective functions and the summation of all deviational variables. The negative sign preceding the deviational variable tries to minimize the deviational variables. By this way equal preference is given to both parts of the final objective function.

**Fig 5:**
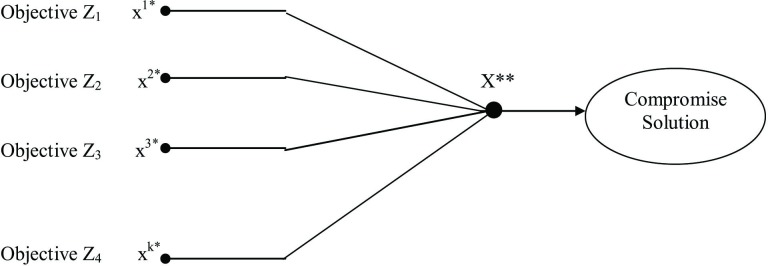
Sketch of the operation of the SICCON technique.

If n is the number of decision variables and k the total number of objectives, then the number of variables for the SICCON Technique totals [n + k (k-1)], where k represents the number of iterations required to find the individual optimal solution. The sketch for the operation of the SICCON Technique is shown in Fig 5.

### Objective functions and calculation procedure

The problem under consideration is how to allocate limited water resources among competing water demand sectors, while maintaining a balance in the system which maximizes the overall system benefits. Therefore, to solve such a problem, a compromise has been established between the upper and lower level decision makers, and the objectives functions are:

$$\left(\mathrm{Upper}\;\mathrm{level}\right)\;\mathrm{Max}\;B=C\sum_{i=1}^{4}x_{i(S)}$$ (5)

$$\left(\mathrm{Lower}\;\mathrm{level}\right)\;\mathrm{Max}\;b_{i}=\sum_{i=1}^{4}d_{i}\cdot x_{i(B)}$$ (6)

In which,

$$x_{S}=\frac{1}{n}\sum_{i=1}^{n}\frac{S_{i}}{D_{nori}}$$ (7)

$$x_{B}=\frac{\sum_{i=1}^{4}S_{i}\times NEB_{i}}{AW_{\left(total\right)}\times NEB_{\max}}$$ (8)

Bi-level water allocation program

$$Maximize\;F_{{}_{i}}=w_{1}\times \left[C_{i}\sum_{i=1}^{4}x_{i}\right]+w_{2}\times \left[\sum_{i=1}^{4}d_{i}\cdot x_{i}\right]-\left(\sigma_{ul}^{-}+\sigma_{ul}^{+}\right)$$ (9)

Subject to:

$$\sum_{i=1}^{4}x_{i}\leq AW$$ (10)

$$x_{inor}\geq x_{i}\geq x_{i\min}$$ (11)

$$\sum_{i=1}^{4}x_{i}\leq \sum_{i=1}^{4}x_{inor}$$ (12)

In addition to the constraints in Eqs (10)–(12), an additional compromise constraint has been developed between the upper level and lower level programs to solve the multi-objective function of the bi-level decision makers, as follows:

$$w_{1}\times \left[C_{i}\sum_{i=1}^{4}x_{i}-\left(B\right)\right]-w_{2}\times \left[\sum_{i=1}^{4}d_{i}\cdot x_{i}-\left(b_{i}\right)\right]+\left(\sigma_{12}^{-}-\sigma_{12}^{+}\right)=0$$ (13)

Where *B* is the benefits of the upper level programming (US$), *b*_*i*_ is the profit of the lower level programming (US$), *F*_*i*_ is the total benefit from the upper and lower level (bi-level) programming (US$), *x*_*i(s)*_ and *x*_*i(B)*_ are the decision variables indicating allocated water to each sector based on the rate of satisfaction and economic benefits, respectively (L^3^), *C* is the value per 1000 cubic meter water (US$/1000 m^3^), *d*_*i*_ is the water price per cubic meter of the allocated water (US$/m^3^), *S*_*i*_ is the amount of water supplied to sector *i* (L^3^), *NEB*_*i*_ is the net economic benefits per unit volume of water from sector *i* (US$/m^3^), *NEB*_*max*_ is the maximum NEB among the sectors considered (US$/m^3^), *AW* is the total available water for allocation (L^3^), *x*_*imin*_ and *x*_*inor*_ are the minimum and normal water demands of each sector (L^3^), $\sigma_{ul}^{-},$$\sigma_{ul}^{+}$ are the negative and positive deviations from the supposed to-be-zero (ideal solution) values of the compromise-constraint between the individual decision makers, and *w*_1_ and *w*_2_ are the weights assigned to the upper and lower level decision makers.

### Net economic benefits to different sectors

The minimum seasonal demand (DS_min_) and the normal demand (DS_nor_) of different water use sectors along with the upper level benefits (ULB) and the lower level benefits (LLB) are given in Table 1. The minimum and normal seasonal demands are obtained by accumulating the minimum and normal monthly demands of each sector during the dry season (Oct-Mar). The value of ULB is the value of water per unit volume of water released from the reservoir for any sector and is constant for each sector [51]. NEB to various water users have been derived from the published studies [52,53]. Using Nayak’s [54] method, the detailed calculations of the NEB for various water-use sectors are given in WAPDA [55] and are briefly discussed in the following paragraphs.

**Table 1:** Seasonal water demands and NEB of various water-use sectors.

Variables	Irrigation	Industry	Domestic	Environment
DS_min_ (Mm^3^)	828	63	45	50
DS_nor_ (Mm^3^)	1283	98	70	100
LLB (US$/10^3^ m^3^)	78	128	412	7
ULB (US$/10^3^ m^3^)	22			

The net economic benefits from water supplied to irrigation are calculated from the total benefits of crop production minus the total production cost and then divided by the total volume of water supplied to the crop. To determine the monthly economic benefits, the seasonal NEB is multiplied by the ratio of monthly water supplied to the total seasonal water supplied while the costs like fertilizer, labor, machinery etc. are considered as constant throughout the month.

$$NEB_{irr}=\sum_{1}^{n}\left[\begin{array}{l} \left\{\frac{\left(A\times Y\times P\right)agdm,cp-A\times \left(F+M+L+O\right)agdm,cp-ws\times \sum_{1}^{m}MW\left(agdm,m\right)}{\sum_{1}^{m}MW\left(agdm,m\right)}\right\}\\ \times \frac{\sum_{1}^{m}MW(m,cp)}{\sum_{1}^{n}MW(m,cp)} \end{array}\right]$$ (14)

Where *NEB*_irr_ is the net economic benefits from the irrigation sector (US$/m^3^), *agdm* is the agriculture demand site, *cp* is the crop, *m* is number of months, *n* is number of crops, *A(agdm*,*cp)* is the area of agriculture for the crop (ha), *Y* is the actual yield of a specific crop (t/ha), *F* is the fertilizer cost (US$/ha), *M* is the machinery cost (US$/ha), *L* is the labor cost (US$/ha), *O* is the other production costs (US$/ha), *P* is the crop price (US$/t), *ws* is the water supply cost (US$/m^3^), and *MW*(*agdm*,*m*) is the monthly withdrawal for irrigation at the take-off level (m^3^).

The water supplied from the reservoir to the residences and the public and other offices in different municipalities is taken as the domestic water use sector and its benefits are estimated by using the inverse demand function [56]. This is calculated as the difference between the water use benefits, minus the installation and maintenance cost of the water conveyance system. This difference is then divided by the volume of water supplied from the reservoir giving the NEB per unit volume of water use, as follows:

$$NEB_{d}=\left[\omega_{0}\left(d\right)\times p_{0}\times \left\{\frac{1}{1+\alpha }\times {\left\{\frac{\omega \left(d\right)}{\omega_{0}\left(d\right)}\right\}}^{\alpha }+\left(0.743-\frac{1}{1+\alpha }\right)\right\}-\omega \left(d\right)\times \omega s\_c\left(d\right)\right]$$ (15)

Where *NEB*_*d*_ is the net profit from the domestic sector (US$/m^3^), *ω*_*0*_(d) is the maximum normal monthly withdrawal of the domestic sector (m^3^), ω(d) is the actual water withdrawal of the domestic sector (m^3^), *p*_0_ is the value of water in the domestic sector at full use (US$/m^3^), *e* is the price elasticity of demand in the domestic sector, α = 1/*e*, and ωs_c(d) is the water supply cost of the domestic sector (US$/m^3^).

The net benefits from the industrial sector are also estimated in the same way as those for the domestic sector, i.e. using the inverse demand function for water. The ratio of the difference between the water use benefits, and the water conveyance system cost to the volume of water supplied from the reservoir, giving the NEB per unit volume of water used. The results of the empirical studies [57,58] can also be used to calculate the net economic returns to water use in industrial and domestic sectors.

$$NEB_{in}=\left[\omega_{0}\left(in\right)\times p_{0}\times \left\{\frac{1}{1+\alpha }\times {\left\{\frac{\omega \left(in\right)}{\omega_{0}\left(in\right)}\right\}}^{\alpha }+\left(0.743-\frac{1}{1+\alpha }\right)\right\}-\omega \left(in\right)\times \omega s\_c\left(in\right)\right]$$ (16)

Where *NEB*_in_ is the net profit of the industrial sector (US$/m^3^), *ω*_*0*_(in) is the maximum normal monthly withdrawal in the industrial sector (m^3^), *ω*(in) is the actual water withdrawal from the industrial sector (m^3^), *p*_0_ is the water value in the industrial sector at full use (US$/m^3^), *e* is the price elasticity of the demand in the industrial sector, α = 1/*e*, and *ωs_c* is the water supply cost for the industrial sector (US$/m^3^).

In this study, the hydropower sector is considered as a non-competing sector as the water released from the reservoir passes through the turbine to generate the hydropower. The NEB of the hydropower sector is computed as the ratio of the hydropower generation multiplied by the difference between the power selling price and the generation cost to the volume of water passing through the power plant, as follows:

$$NEB_{p}=\frac{Power(pwst)\times \left[P_{price}\left(pwst\right)-P_{\cos t}\left(pwst\right)\right]}{Q_{total}}$$ (17)

Where *NEB*_p_ is the net profit from the power production (US$/m^3^), Power(pwst) is the power produced at the production site (kWh), *P*_price_, is the average power selling cost (US$/kWh), *P*c_ost_(pwst) is the average power production cost (US$/kWh), and *Q*_total_ is the total volume of water passing through the plan (m^3^).

As there is no well-established method available to calculate the exact net economic benefits in the environment water sector, therefore the benefits from this sector are calculated as the costs of avoiding damages or replacing services of the infrastructure which can cause due to the salt water intrusion. The water allocation for this sector is mainly to control the saltwater intrusion.

### Model application

**Fig 6:**
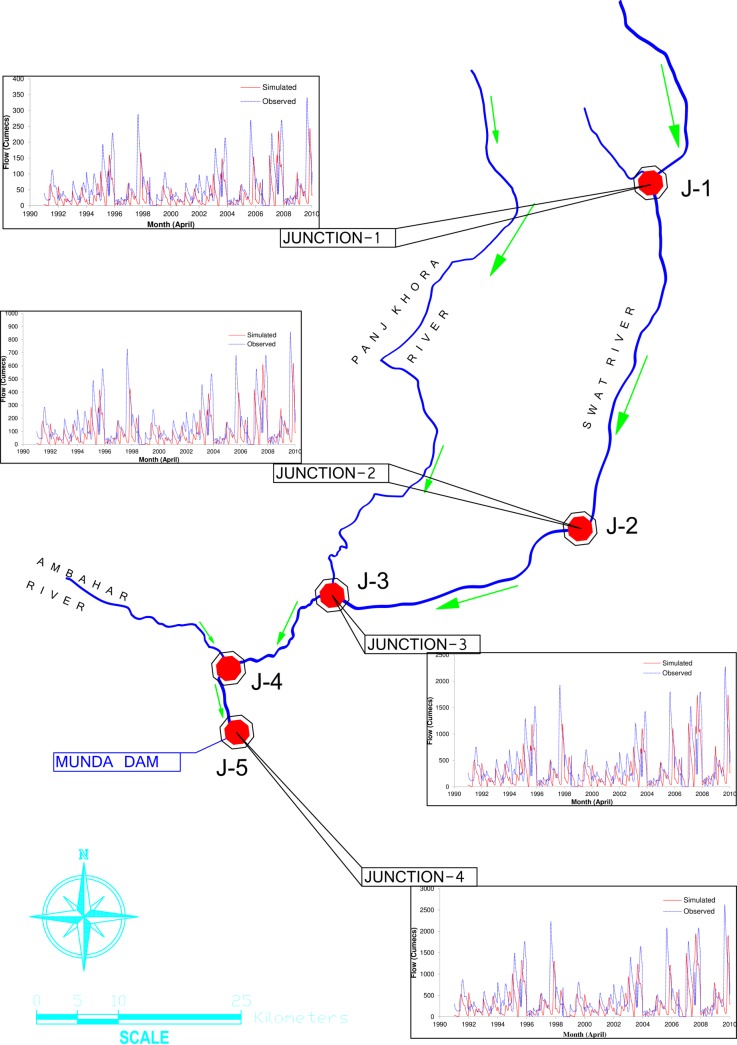
Stream network and direction of flow in Swat River basin.

The developed BLMOLP model has been applied to the Swat River in Khyber Pakhtunkhwa province of Pakistan. The input data for reservoir operation model (ROM) includes the monthly inflows into the reservoir, the reservoir physical characteristic (i.e. area-elevation-storage relationship) and the reservoir operating rules, rainfall and evaporation, percolation, channel characteristics and monthly water demand of the various water users. HEC-HMS model was run on the daily basis and the simulation has been carried out for a period of 20 years (i.e. 1 Jan 1990 to 31 Dec 2010) to estimate the inflows at Munda Dam site of the Swat River basin. The observed inflow data are presented in S1 Table. Fig 6 shows the river network of Swat River basin and the comparisons between the observed and model simulated flows. A combination of manual and automated techniques was used for the calibration process in the HEC-HMS model. In automated calibration techniques the model calculates the optimized parameter values that could result the best fit between observed and simulated runoffs [59]. A good agreement between the observed and simulated flows was found at the Junction J-1, where the Gabral and Ushu rivers join the Swat River. At Junctions J-2 (Chakdara), J-3 (Zulam Bridge) and Junction J-5 at Munda reservoir, the simulated and observed flows were reasonably comparable as well.

After incorporating the reservoir losses and gains in the ROM, the calculated inflows by the model have been considered as the AW, which is then used as the input in the BLMOLP. As stated earlier, the available water in the dry season is less than the total water required to satisfy the demand of various water use sectors. The water demands of the various sectors have been pooled sector-wise and considered for water allocation. To demonstrate the model applicability, the limited available water resources of 359, 93, 81, 130, 172 and 429 Mm^3^ for the months October to March in the dry season have been allocated for the monthly minimum and normal demands (DM_min_ and DM_nor_). As a solution to the bi-level water allocation problem, the amount of water allocated (WA_m_) is between the minimum and normal demands. The normal demand of a particular sector is defined as the water demand which that sector needs to fulfill its water requirements, and the minimum demand is the amount of water which must be released to the sector to fulfill its minimum requirements.

In solving the multi-objective function, equal weights (*w*_1_ = *w*_2_ = 0.5) have been assigned to the upper and lower level DMs. However, the weights can be varied depending on the priority either given to the upper or lower level DMs.

## Results and discussions

### Equity based water allocation

The BLMOLP model has been applied to the Swat River basin in Khyber Pakhtunkhwa province of Pakistan, for optimal allocation of limited water resources to four competing water use sectors: i.e. irrigation, industry, domestic, and environment. Further, the hydropower sector is not considered as a competing water demand sector because the water released to various sectors passes through the turbines for hydropower production. Therefore, hydropower is produced as a by-product. By assigning equal priorities to all sectors, Table 2 shows the detailed model results in allocating the limited water resources among competing water demand sectors in different months of a season. The model performance is satisfactory in the monthly water allocation program as the water allocated (WA_m_) is between the minimum and normal demands of each sector in each month, as shown in Table 2.

**Table 2:** Monthly water allocation results with equal priorities assigned to each sector.

Sector	Variables	Oct	Nov	Dec	Jan	Feb	Mar	Total/ average
Irrigation	DM_min_ (Mm^3^)	244	56	35	78	140	274	828
	DM_nor_ (Mm^3^)	379	87	55	121	217	425	1283
	WA_m_ (Mm^3^)	312	64	48	101	149	381	1054
	Satisfaction level (%)	82	73	88	83	69	90	81
	NEB_m_ (US$×10^6^)	31	6	5	10	15	38	105
Industry	DM_min_ (Mm^3^)	16	10	8	7	7	15	63
	DM_nor_ (Mm^3^)	25	15	12	11	12	22	98
	WA_m_ (Mm^3^)	20	11	11	10	8	20	80
	Satisfaction level (%)	82	74	89	83	65	90	80
	NEB_m_ (US$×10^6^)	3	2	2	1	1	3	12
Domestic	DM_min_ (Mm^3^)	11	7	6	6	6	10	45
	DM_nor_ (Mm^3^)	17	11	9	9	9	15	70
	WA_m_ (Mm^3^)	14	8	8	8	6	14	57
	Satisfaction level (%)	82	74	89	83	65	90	80
	NEB_m_ (US$×10^6^)	6	3	3	3	3	6	25
Environment	DM_min_ (Mm^3^)	8	8	8	8	8	8	50
	DM_nor_ (Mm^3^)	17	17	17	17	17	17	100
	WA_m_ (Mm^3^)	13	10	14	13	9	14	73
	Satisfaction level (%)	75	62	84	76	56	85	73
	NEB_m_ (US$×10^6^)	0.4	0.3	0.4	0.4	0.3	0.4	2
Hydropower	Water through turbine	346	83	67	118	163	414	1191
	NEB_m_ (US$×10^6^)	9	2	2	3	4	11	32
**Total Benefit (US$×10**^**6**^**)**		**50**	**14**	**12**	**18**	**23**	**58**	**176**

As the upper level DMs tend to distribute the water resources to the lower level water users based on the equity system, therefore, the upper level controls the water allocation program as it aims to provide sustainability to the water allocation program. On the other hand, the lower level DMs try to maximize the individual sectoral benefits. When equal priorities are given to each water demand sector, the water shortage is distributed equally to all the sectors. When the stress is equally distributed among all the sectors in a water scarce season, each of the water demand sectors receive water less than its normal demand but can survive since its minimum demand is fulfilled. Table 2 shows that all the sectors receive more water than their minimum requirements but less than the normal demands.

**Fig 7:**
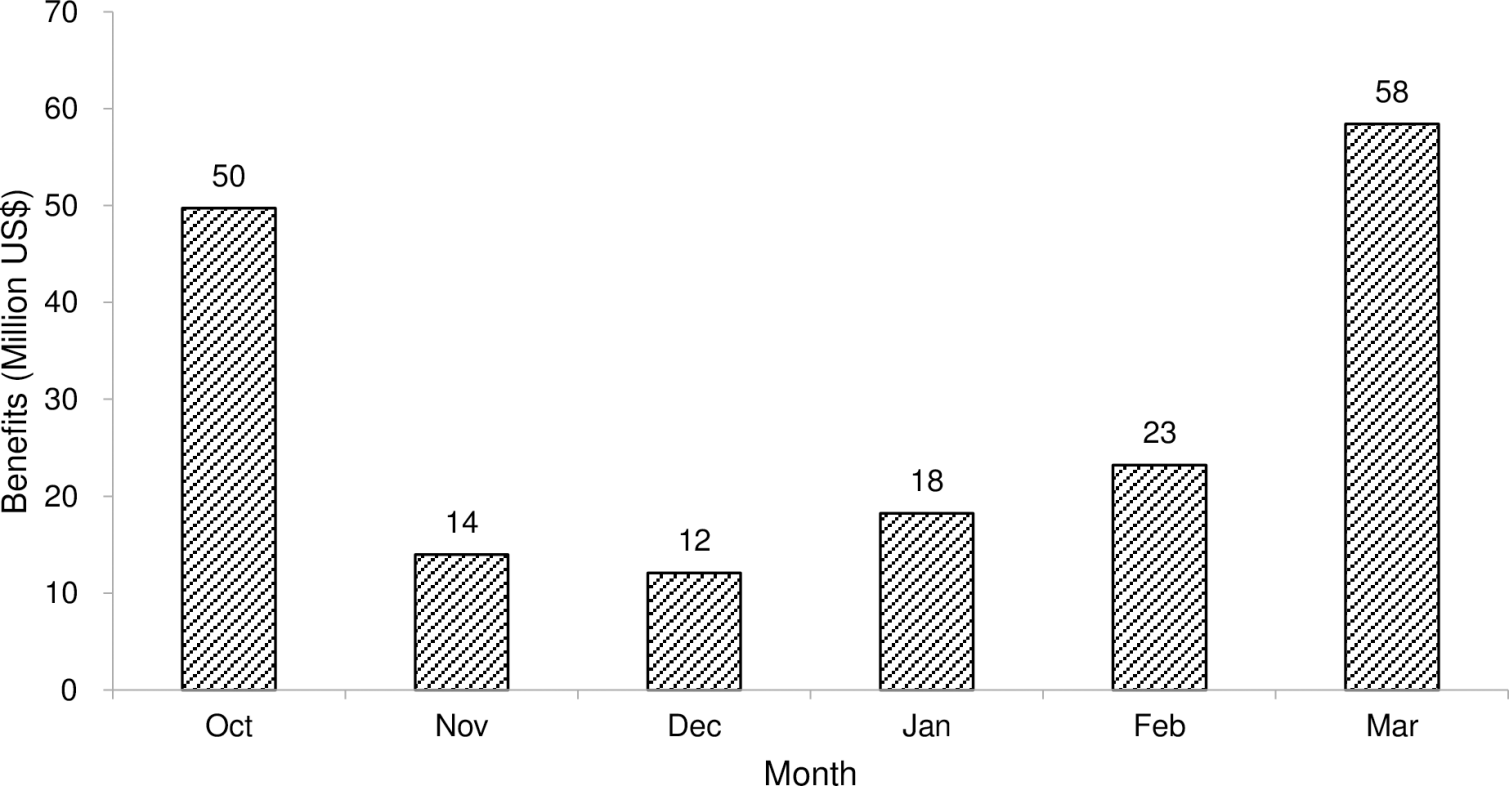
Total benefits by all water users in different months based on equity.

The satisfaction rate of a particular water use sector is the ratio of the amount of water supplied to the normal demand by that sector in a selected month or season. The level of satisfaction is an indication of the percentage of the water demand fulfilled; the remaining percentage is the stress level. When equal preference is given to all sectors, none of the sectors is fully satisfied and the water shortage is equally distributed among the sectors. The accumulated net economic benefits of all the water demand sectors in different months of a season are shown in Fig 7. In March, the maximum economic returns are the maximum amount of water allocated to various sectors in this month because the accumulated water demand of all the sectors is highest as compared to that in the other months for the selected season. The total value of the net economic returns in the whole season from all the sectors including the upper level benefits is US$176 million and the average satisfaction level is 79%.

### Priority based water allocation

In most of the practical cases, the priorities are different depending on the local conditions and social preferences, e.g. in a developing country like Pakistan the priority could be the agriculture sector and the maximization of economic benefits can be tolerated and vice versa [16]. Therefore, different flexible scenarios have been developed by assigning priorities to the water demand sectors to evaluate the model applicability under different conditions. As in some of the regions or areas, irrigation could be the prioritized sector, therefore the upper level decision makers try to maximize the water allocation for the irrigation sector. However, in other regions, domestic sector maybe the priority in order to maximize the economic returns. Therefore, the priority is different for different area depending on the requirements of that particular area.

In this study, four scenarios have been developed by assigning priorities to different water demand sectors, namely: irrigation, industry, domestic and environment. The developed scenarios give a wide spectrum of the situation to the local decision makers and allow them to further develop and analyze other scenarios which suit the situation and improve the water management in the water scarce regions. The summations of the monthly minimum and normal water demands (DM_min_ and DM_nor_) give the seasonal minimum and normal water demands (DS_min_ and DS_nor_). The model has been applied to allocate water on a seasonal basis. The input parameters and results of the developed scenarios are compared and shown in Table 3, which are discussed in subsequent sections.

**Table 3:** Comparison of water allocations in four scenarios.

Sectors	Input Data			Scenario-I: Irrigation priority			Scenario-II: Industry priority			Scenario-III: Domestic priority			Scenario-IV: Environment priority		
	Net economic benefits (US$/10^3^ m^3^)	Seasonal Demands (Mm^3^)		Allocated water (Mm^3^)	Level of satisfaction (%)	Total Benefits (US$×10^6^)	Allocated water (Mm^3^)	Level of satisfaction (%)	Total Benefits (US$×10^6^)	Allocated water (Mm^3^)	Level of satisfaction (%)	Total Benefits (US$×10^6^)	Allocated water (Mm^3^)	Level of satisfaction (%)	Total Benefits (US$×10^6^)
		DS_min_	DS_nor_												
Irrigation	78	828	1283	1086	85	109	1039	81	104	1044	81	104	1031	80	103
Industry	128	63	98	70	71	10	98	100	15	79	80	12	78	79	12
Domestic	412	45	70	50	71	22	56	80	24	70	100	30	55	79	24
Environment	7	50	100	59	59	2	71	71	2	72	72	2	100	100	3
Hydropower	5	936	1205	1205	100	32	1193	100	32	1192	100	32	1164	100	31
**Total/** **average**		**986**	**1551**	**1264**	**71**	**174**	**1264**	**83**	**177**	**1264**	**83**	**180**	**1264**	**85**	**172**

#### Scenario-I: Irrigation priority

In this scenario, priority is given to the irrigation sector in order to maximize the water allocation to this sector. In this case, the upper level DMs allocate the maximum amount of water to this sector while also making sure that the minimum water requirements of the other sectors are fulfilled. The model first fulfills the minimum requirements of all the sectors. After that, water is allocated according to the given priority, such as the irrigation sector. Then, the remaining water is allocated to the other sectors. Table 3 shows the results of the BLMOLP model application with the assigned priority to the irrigation sector. Table 3 shows that the level of satisfaction of irrigation sector is 85% as compared to 81% when equal priorities are given to all the sectors. Also, the value of the total economic benefits produced by the irrigation sector has increased from US$105 million to US$109 million.

**Fig 8:**
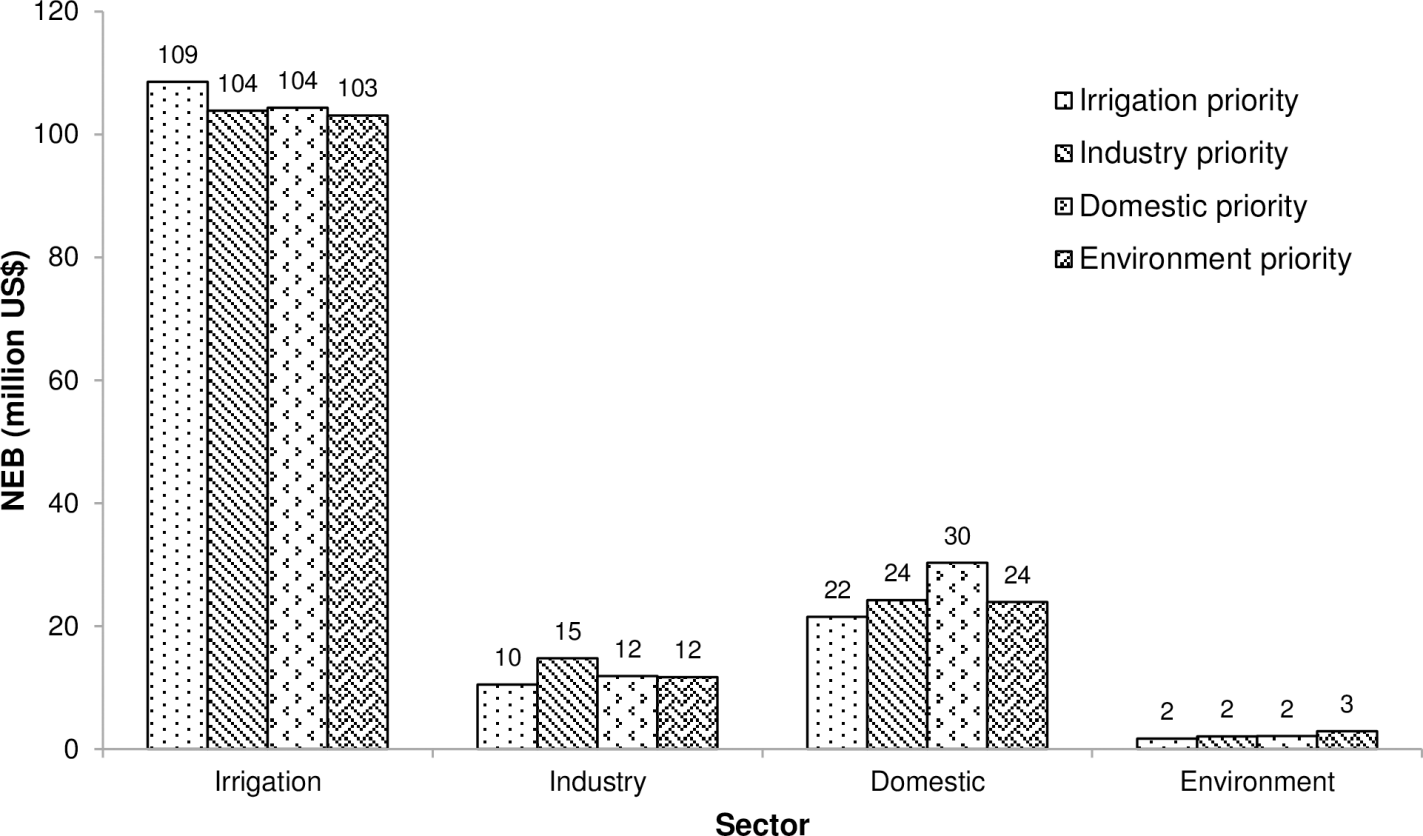
Comparison of economic benefits in different scenarios.

The satisfaction level for the hydropower sector is 100% in all the scenarios as it is not a competing water demand sector. In this Scenario, the value of the total economic returns by all the sectors including hydropower is US$174 million. Fig 8 shows that in Scenario-I, the economic benefits produced by the irrigation sector is the highest, as compared to all the other developed scenarios. Furthermore, the economic benefits in this scenario are less than the benefits produced when the equal priority is given to all the sectors (Table 2) as the economic value of irrigation water is less than those of the industrial and domestic sectors.

#### Scenario-II: Industry priority

In Scenario-II, the priority is given to the industrial sector for the optimal allocation of water resources based on bi-level programming. As the upper level decision makers control the water allocation program by releasing the water to lower level water users. Therefore, in this scenario, the water is first allocated to the industrial sector. The performance of the BLMOLP model is also satisfactory in this scenario as all the sectors fulfill their minimum water requirements. As the normal water demand is met, the level of satisfaction of the industrial sector is 100% as compared to 80% when the model operates based on the equity system. In this scenario, the economic benefits to the industrial sector are the highest as compared to the other scenarios, as shown in Fig 8. Moreover, the value of the total economic benefits produced is US$177 million, which is more than the economic benefits when equal priorities are assigned to all the water demand sectors. This is because the economic value of the industrial water use is higher than those of the irrigation and environment sectors (Table 2).

#### Scenario-III: Domestic priority

A third scenario has been developed by assigning priority to the domestic sector and its results are shown in Table 3. Same as in Scenario-I and Scenario-II, the BLMOLP model first allocates water to fulfil the minimum water requirements of all the sectors. After that, the model allocates the water according to the assigned priority. As the prioritized sector in this scenario is the domestic sector, the model allocates the remaining water to this sector to meet its normal demand. After meeting the normal demand of the domestic sector, if there is any water left, then the model allocates the water to the sector which has minimum water requirements and maximum economic benefits. In this way, the maximum economic returns are achieved with minimum water release.

As expected for this scenario, the satisfaction level for the domestic sector is 100%. Hence, the domestic sector produced the maximum economic benefits in Scenario-III as compared to other scenarios, as shown in Fig 8. The value of the total economic benefits produced by all the water demand sectors is US$180 million, which is the highest among all the scenarios. Table 2 shows the total economic benefits when equal priority has been assigned to all the sectors.

#### Scenario-IV: Environment priority

In this scenario, priority has been given to the environment sector. A certain amount of water is required to be released to the downstream river for salinity control and to protect the downstream river inhabitants. In some regions or areas, the environmental sector maybe the prioritized sector in order to maximize the economic returns by protecting the downstream river inhabitants and by salinity control, which could otherwise damage the river infrastructure installments. In this study, the benefits from the environment sector are considered as the cost which could be saved by preventing the damages caused by salinity. Table 3 shows the results when priority is given to the environment sector.

Fig 8 shows that the value of the benefits derived from the environment sector is the highest as compared to the other sectors. Moreover, of the satisfaction level for the environmental sector is 100% and the value of the total benefits from this sector in Scenario-IV is US$5 million. In this scenario, the value of the total economic benefits produced by all the sectors is US$172 million which is less than the total benefits produced when equal priorities have been assigned to all the sectors (Table 2). This is because the unit value of benefits from the environmental sector is the lowest among all the considered water demand sectors.

## Discussions

Bi-level programming issues are frequently found in the allocation of water resources among various water users [30]. The proposed model offers an insight into the economic, water supply and hydrologic interaction for water allocation to distinctive water users. In the present study, there are not only conflicts among the different water users but also between the water users and reservoir managers. Consequently, BLMOLP was evolved to optimally allocate the water resources among competing water users for sustainable economic development. The developed BLMOLP model is applied to a single reservoir by aggregating the releases from the reservoir for water allocation. However, the model structure may be improved by way of integrating parallel stems and can be applied to complex water resource networks or the model can be run separately for each reservoir in the network. The reservoir is fulfilling the irrigation water demands of the areas located right away downstream, however, the water shortages occur in the further downstream areas in the course of the dry seasons.

The reservoir simulation and water allocation calculations were performed for a period of 20 years. However, the water allocation results are only shown for a dry season (Oct-Mar) with the AW of 1264 Mm^3^ against the total normal demand of all water users of 1591 Mm^3^. In this study, water is allocated based on the equity based and priority-based systems. Equal priorities were assigned to each water user in the equity-based system, which is also the current water allocation practice. In the priority based system, different scenarios were evolved by assigning priorities to specific water users to illustrate the model applicability under different conditions so that a suitable scenario acceptable to stakeholders may be developed, analyzed and implemented objectively to the water situation in the basin [16].

When the equal priorities were assigned to each sector, BLMOLP maximizes the equity in the water allocation system and the maximization of economic returns of individual sectors maybe compromised. Therefore, in a water allocation system based on equal priorities optimizes the upper level decision making process but it might compromise the decisions by the lower level decision makers. In priority based water allocation system, when a sector given priority produces highest economic benefits and satisfaction rate among all the scenarios because the model first allocates the water to the prioritized sector then to the remaining sectors based on their demands and net economic returns and the results were found consistent with earlier studies [30,60] under these conditions.

When a particular water user gains priority, it attains maximum economic returns and satisfaction level among all the other selected water users and these results were found consistent with the previous studies using SICCON technique in water allocation. Using the Gini coefficient, a power index and economic efficiency function, Hu et al. [38] determined that whenever the AW to a water user is rises, satisfaction level and economic benefits to that user also increases under a bi-level water allocation problem. The results of the current study are found consistent with [61], in which a two-stage regional multi-water source allocation model was developed which was capable to optimize the water allocation framework for water resources managers and DMs along with the benefits for the individual water users. Furthermore, similar results were found in a two-stage stochastic fractional programming (TSFP) method for planning of an agricultural water resources management system [62]. However, the techniques used in the present study are mathematically simple and easy to apply and, therefore, technology transfer is considered to be more effective.

There are some limitations and challenges that need to be addressed in the water allocation model developed in this study through further investigations. NEB to water use for salinity control and navigation should be determined using other suitable approaches and methods instead of the replacement cost method adopted here. The study assumes a fixed net benefit irrespective of amount of water allocated to each of the use sectors. This is justified as the monthly variation in the water allocated to different sectors is very small [60]. Moreover, the weights given to different water users can affect the results significantly, however, this study considers the same weight to different water users for simplicity in allocating the water resources which is not realistic as different water users may have different priorities depending upon their importance in the society irrespective of the economic returns [16,48]. Therefore, in future studies varying weights maybe assigned to different water users depending upon the objective functions and their importance in the society when designing the water allocation system for a reservoir.

Additionally, to address complex water resources network the two components (ROM and BLMOLP) of the model along with the hydrologic uncertainties should be combined and allocation be made at spatial locations in the river basin to take full advantage of an integrated hydro-economic model which runs with results of ROM fed into BLMOLP and the results of BLMOLP into the ROM until the optimized allocation of water with maximized economic benefit is achieved.

## Conclusions

In this study, a bi-level multi-objective model has been developed for the optimal water allocation under the heirachical structure. The model consists of a ROM and a BLMOLP. The ROM estimates the AW for allocation in a dry seasoan, which is used as an input to the BLMOLP. The BLMOLP model allocates the AW based on the decisions made by the upper level DMs (i.e. leaders) and the lower level DMs (i.e. followers). The model has been applied to the Swat River basin of Pakistan for an optimal allocation of AW among competing water use sectors, i.e. irrigation, industry, domestic and environment. Different techniques have been used to estimate the NEB to water use in irrigation, domestic, industrial, hydropower, and environmental (salinity control) sectors. The NEB is as low as USD 5 per thousand m^3^ for hydropower sector to as high as USD 412 per thousand m^3^ for domestic use. The estimated NEB of water use in the agriculture sector is USD 78 per thousand m^3^. The environmental (salinity control) sector has a NEB of USD 7 per thousand m^3^.

The study analyzes the performance of developed water allocation model under two conditions, i.e. by assigning equal preference to all the sectors, and prioritizing individual water user. When equal priority is given to all the water demand sectors, the water allocated to each sector do not meet the normal demand of any sector because the AW is less than the total demand of all water users. However, the minimum water requirements of all the sectors are accomplished. The value of the total NEB from all the water users is US$176 million and the average satisfaction level is 79%. Furthermore, four scenarios have been developed by prioritizing the four water use sectors individually, i.e. irrigation, industry, domestic and environment. The model results show that for the four scenarios, the economic returns are US$174, 177, 180 and 172 million, respectively.

The bi-level programming model developed in this study provides a higher motivation for water saving and alleviates the conflict between water demand and supply by introducing the concepts of satisfaction rate and economic benefits together. Also, the BLMOLP model has the advantage in addressing the bi-level water allocation problem because of its fewer requirements for data collection and solution generation.

## Supporting information

S1 Table Daily inflows data. (XLSX)

## Abbreviations

BLMOLP: bi-level multi-objective linear program
DMs: decision makers
SICCON: simultaneous compromise constraint technique
WT: weighting technique
AW: available water
ROM: reservoir operation model
m^3^/s: cubic meter per second
Mm^3^: million cubic meter
NEB: net economic benefits
DS_min_: minimum seasonal demand
DS_nor_: normal seasonal demand
DM_min_: minimum monthly demand
DM_nor_: normal monthly demand
